# Evaluation of migration analysis with AI-based CT-RSA and preoperative 3D-planning in total hip arthroplasty

**DOI:** 10.2340/17453674.2025.44948

**Published:** 2025-12-11

**Authors:** Albin CHRISTENSSON, Hassan M NEMATI, Kristina YDSTRÖM, Gunnar FLIVIK

**Affiliations:** 1 Department of Orthopedics, Skåne University Hospital, Clinical Sciences, Lund University, Lund; 2 Ortoma AB, Gothenburg; 3 Radiation Physics, Department of Hematology, Oncology and Radiation Physics, Skåne University Hospital, Lund, Sweden

## Abstract

**Background and purpose:**

Computed tomography (CT) has become a valuable tool for preoperative planning and perioperative, real-time navigation during total hip arthroplasty (THA). CT can also quantify postoperative implant migration without the need for implanted bone markers, making it a promising alternative to the current gold standard radiostereometric analysis (RSA). Our aim was to evaluate the accuracy of preoperative planning and postoperative implant migration of both cup and stem employing AI-based software using 3D CT-images (CT-RSA) compared with conventional RSA.

**Methods:**

26 patients with primary THA were preoperatively 3D-planned and perioperatively navigated. They were followed and analyzed with AI-based CT-RSA within 2 days postoperatively and at 3, 12, and 24 months. 10 of the patients had implanted tantalum markers at surgery and were also followed up with conventional model-based RSA (MBRSA). The results were compared with CT-RSA. Prosthetic CAD models were used for both conventional RSA and AI-based CT-RSA analysis. Double CT and MBRSA scans were taken to evaluate precision. The preoperative plan was compared with actual perioperatively chosen implants.

**Results:**

AI-based CT-RSA showed consistent migration patterns, with most migration in the first 3 months, which then levelled out. Bland–Altman plots indicated good agreement between MBRSA and AI-based CT-RSA. Overall, there was high correspondence between MBRSA and AI-based CT-RSA in translations, but more divergent rotation results. AI-based CT-RSA precision was consistently slightly better than MBRSA precision. The agreement between planned and actual size of cup was 25 out of 26, and 23 out of 26 for stems.

**Conclusion:**

AI-based CT-RSA demonstrated accuracy comparable to MBRSA, with slightly improved precision and reduced user-dependence. The same system also provided an accurate and predictable preoperative implant plan.

Restoring native femoral offset [1-3], leg length [3], and correct acetabular cup orientation improve functional outcome in total hip arthroplasty (THA) and may reduce cup polyethylene wear [4,5]. Preoperative planning allows for better implant sizing and position, thus achieving reproducible results [6]. Conventional templating on 2D radiographs has limitations, as individual patient anatomy can be misrepresented, for example, by femoral rotation [7]. In contrast, 3D computed tomography (CT)-based planning is more accurate and may enhance reliability [8].

The use of CT has been limited due to high radiation doses. With the advancement of low-dose technology, CT has, however, become available, not only for THA templating [9], but also for measurement of postoperative implant migration in the bone over time [10]. For CT-RSA dedicated computer software is needed, but it does not require perioperative placement of bone markers for migration analysis [11], or examination equipment specific for conventional RSA [12]. Previous studies have shown promising results for CT-RSA replacing conventional RSA [13-17]. Available CT-RSA systems still require manual and time-consuming procedures, including bone segmentation and image registration similar to conventional RSA. With the use of artificial intelligence (AI), images can be processed automatically with high accuracy reducing time requirement [18].

In this study, we have evaluated a novel computer-assisted orthopedic surgical platform that consists of AI algorithms and machine learning (ML) models to automate segmentation, landmark localization, 3D reconstruction, and image registration. The software platform contains 3 modules: Hip Plan, Hip Guide, and Follow-up. We have previously shown that the Hip Guide offers intraoperative navigation guidance of good precision in cup placement [19] and that the Follow-up tool (AI-based CT-RSA) is an alternative to model-based RSA (MBRSA), even though prosthetic CAD models were not implemented in the software [20].

We aimed to analyze the migration results, with repeated measurements up to 2 years, in an uncemented THA obtained from AI-based CT-RSA. In a subgroup we directly compared the results between CT-RSA and MBRSA and have also included double examinations for precision evaluation. In addition, we assessed the accuracy of the preoperative planning tool using the same CT scans.

## Method

### Study design

Our inclusion criteria were patients between 40 and 75 years of age with primary or secondary coxarthrosis planned for uncemented THA. Exclusion criteria were BMI > 35, inability to assimilate the patient information, and other reasons causing the surgeon to judge the patient as unsuitable, e.g., serious illnesses or addiction.

All surgeries were performed at Skåne University Hospital, Trelleborg site, Sweden, by an experienced hip surgeon (GF). Patients received a cementless Corail stem and Pinnacle cup (Johnson&Johnson MedTech, Warsaw, IN, USA) with instrumentation according to the manufacturer’s instructions using a posterior approach.

The hydroxyapatite coated stem comes in a variety of sizes, including a short neck, high offset, and a coxa vara option to restore individual patient anatomy. Both a collarless and a collared alternative are available. In this study, the collared stem was used.

The study is reported according to STROBE guidelines.

In this study, we have used the Ortoma Treatment Solution (OTS, Ortoma AB, Gothenburg, Sweden). It contains 3 modules: Hip Plan, Hip Guide, and Follow-up. The Hip Plan and Hip Guide modules have FDA clearance and are MDR CE and JP certified. The Follow-up module is MDR CE and JP certified.

### Preoperative 3D planning and perioperative navigation

Before surgery all patients underwent a low-dose CT scan of the pelvis, with complementary views of knees and ankles in order to measure leg length and neck anteversion. Preoperative templating was done in 3D, using the OTS Hip Plan. The software employs AI to identify landmarks and suggests suitable implant sizes and positions. The surgeon then has the opportunity to make final adjustments before approving the surgery plan. The OTS Hip Guide was used intraoperatively, with tracers mounted on instruments and fixed to patient bone, in order to navigate the implants into the preoperatively planned position, yet with the ability to make changes based on the intraoperative situation. The AI-planned cup and stem size, adjusted when needed by the surgeon, was compared with the definitive implant size. More information on the OTS Hip Plan and Guide tool with precision evaluation has been published in a previous paper [19].

### AI-based CT-RSA

After surgery, a low-dose CT scan (pelvis, knees, and ankles) was performed within 2 days after surgery and then at 3 months, and 1 and 2 years postoperatively. The data was analyzed with the OTS Follow-up, AI-based CT-RSA. The software does not use tantalum markers for the migration analysis. Instead, it relies on automatically generated bone and implant segmentation, and anatomical landmarks from its integrated ML models. The ML models are developed through the use of Convolutional Neural Networks (CNNs), a specific subset of ML that focuses on neural networks featuring convolutional layers. The training and test data used for developing the CNNs consists of CT from hip patients including more than 140.000 images. The CNNs are trained, evaluated, and tested thoroughly to ensure integrity and generalizability.

AI-based CT-RSA analyzes migration between 2 rigid bodies between 2 CT examinations. To do so, the following steps were performed. The postoperative, 3 months, 1- and 2-year CT images of each patient were imported, and the software performed automatic segmentation and landmark localization on each CT image series, resulting in the segmentation and localization of bones (pelvis and femur) and implants (cup and stem). The femoral or pelvic bone was defined as the fixed reference. The software automatically aligns the reference bones in the subsequent scans as closely as possible using a rigid alignment algorithm. The potentially migrating part (stem or cup) was detected, and the prosthetic CAD model fitted to the implant. Cup and stem CAD models for the analysis were received from the manufacturer. In the final step, the migration of the implant relative to the reference segment was obtained in 6 degrees of freedom (translation and rotation in x, y, and z direction). The software calculates the results automatically, minimizing the need for manual intervention while enabling the clinician to maintain full control over the procedure. All these steps were performed in approximately 5 minutes. In order to run the software, a standard computer equipped with a dedicated NVIDIA graphic processing unit (GPU) with 4 GB memory and NVIDIA GPU computing capability greater or equal to version 6.1 is required.

Patients were scanned with CT using different clinical scanner systems (Philips IQon [https://www.philips.co.uk/]/Siemens Somatom Flash/Siemens Somatom Edge [https://www.siemens-healthineers.com/]) depending on availability. Scanning (for the majority of patients) was done with the same low-dose protocol set up for the study and adopted to patient size using automatic exposure control in order to receive constant image quality (resulting in typical median radiation doses of 2.5 mSv for the pelvis). The setting for pelvis was 120 kV with a reference mAs of 30. For knee and foot, the mAs setting was constant, set at 30 mAs. At some timepoints, another protocol with pelvic fixed mAs (30 mAs) was used to keep the dose lower (typical median radiation < 1mSv). Reconstruction of thin slices (1 mm with 50% overlap) was done with soft filter and noise reduction with iterative reconstruction level 2. Metal artifact reduction (MAR reconstruction) was applied. The estimated effective dose was calculated using conversion factors (ICRP 103) [21].

### Conventional model-based RSA (MBRSA)

10 (of the 26) patients had perioperatively placed bone markers, intended for additional postoperative MBRSA follow-up. The proximal femur and the periacetabular bone were marked with 9–10 tantalum markers (size 0.8 mm) each, aiming for a maximal spread of markers in all directions in the bone in order to get as good a segment for RSA analysis as possible. Thus, 10 patients had MBRSA examinations on the same day as all their CT follow-up scans.

RSA radiographs were taken according to the standard procedure in supine position with a uniplanar calibration cage (Model 43, Tilly Medical, Lund, Sweden) and 2 simultaneously exposed digital radiographs [12]. RSA data was analyzed by an experienced user with the Model-based RSA 4.2 (MBRSA) software (RSAcore, Leiden, The Netherlands). CAD models provided by the implant manufacturer were used for the stem and cup, and we analyzed the migration between CAD model and bone markers. Migration was analyzed along the 3 axes in an orthogonal coordinate system: x, y, and z. The results were recalculated and presented as if all hips were right-sided. Thus, positive directions for translations were medial, superior, and anterior translation along the x-, y-, and z-axes respectively. Positive directions for rotations were anterior tilt (x-axis), internal rotation/anteversion (y-axis), and valgus for stem and decreased inclination for the cup (z-axis). The same coordinate system was used for AI-based CT-RSA. Total translation and rotation were calculated by using the Euclidean formula √(x^2^+y^2^+z^2^). We accepted a conditional number of < 120 (indicating the spread of tantalum markers), and a mean error of rigid body fitting (stability of the markers between 2 examinations) of < 0.35 [12].

The precision of both MBRSA and AI-based CT-RSA was determined by double examinations, i.e., 2 scans of the patient with intermediate reposition. As the implants were not expected to have moved in such a short time, the measurement error was defined as the precision of the system.

To make the migration results between AI-based CT-RSA and MBRSA comparable, we used an implant-based coordinate system for the stem with y-axis parallel to the stem long axis, and x-axis in the same plane as the stem and neck axis, perpendicular to the y-axis. For the cup, a similar implant-based coordinate system was more difficult to achieve because of its spherical shape. For cup comparison, we used the coordinate system defined by the calibration cage for MBRSA [12] and patient position on the CT-table for AI-based CT-RSA.

### Clinical evaluation

All patients were asked to fill in a patient-reported outcome measures (PROM) questionnaire, the Forgotten Joint Score (FJS) [22]. The questionnaire was collected preoperatively, at 3 months, and 1 and 2 years postoperatively.

### Statistics

The postoperative AI-based CT-RSA and MBRSA migration pattern is presented with mean values and 95% confidence intervals (CI). Stem subsidence and retroversion, and cup proximal migration and change of inclination, as well as total translations, were considered as the clinically most important migrations.

Visual analysis of the migration data showed that the majority of migration occurred up to 3 months and then levelled out. Therefore, migration over time was analyzed using a piecewise linear mixed-effect model [23] with breaking point at 3 months. The models included 2 main fixed effects: time starting from surgery, and time starting from 3 months after surgery. Subject was included as a random effect. This enabled us to compare the migration slopes before and after the breaking point.

We graphically assessed agreement between MBRSA and AI-based CT-RSA with Bland–Altman plots. The difference between MBRSA and AI-based CT-RSA for each patient is plotted on the y-axis and the average of the same measurements on the x-axis. Limits of agreement (LoA) were calculated around the mean (mean difference ± 1.96 x SD) [24]. As all patients have a baseline prosthetic position postoperatively, which is then compared with the position at 3 months, and 1 and 2 years after surgery, each patient will contribute 3 migration comparisons, MBRSA vs CT-RSA. Normality of the difference between MBRSA and AI-based CT-RSA was verified by visually inspecting Q–Q plots and histograms.

To further investigate the agreement between conventional RSA and AI-based CT-RSA, repeated-measures mixed models including the 10 subjects with measurements from both methods were performed. From these models, least square means and difference in least square means with corresponding 95% confidence intervals were calculated for 3, 12, and 24 months after surgery.

MBRSA and AI-based CT-RSA precision was calculated as the standard deviation (SD) of the difference between double examinations times the critical value of t for a 2-tailed test, significance level of 0.05, and degrees of freedom based on the number of double exams.

For PROM evaluation, we used the Wilcoxon signed-rank test for paired comparisons.

The implant size from the planning tool was compared with the actual stem and cup size chosen by the surgeon during surgery. The Clopper–Pearson method was used for calculating the binomial proportion confidence interval.

Statistical analyses were performed using IBM SPSS Statistics Version 29.0.2.0 (IBM Corp, Armonk, NY, USA) and SAS/STAT software version 15.2, 2020 (SAS Institute Inc, Cary, NC, USA).

### Ethics, registration, data sharing plan, funding, and disclosures

The project was approved by the Swedish Ethical Review Authority 23/11/2020 (Dnr 2020-03382) and registered in Clinical Trials.gov (NCT05159206). The study was carried out in compliance with the Helsinki Declaration and is reported according to the GRRAS guidelines [25]. Written informed consent was received before inclusion and complemented for patients with both CT and MBRSA follow-up. Data is available on reasonable request. The first author (AC) has received an unconditional research grant supporting the PhD project from Ortoma and the surgeon (GF) has done consultancy work for Ortoma. Johnson&Johnson MedTech has sponsored implementation of the CAD models. HN is employed by Ortoma AB. Complete disclosure of interest forms according to ICMJE are available on the article page, doi: 10.2340/17453674.2025.44948

## Results

### Patients

26 THA patients (6 females), with a mean age of 60 (SD 9) years, were included between November 2020 and May 2022. Patient demographics are summarized in Table 1.

**Table 1:** Patient demographics for all patients (CT-RSA) and subgroup (CT-RSA and RSA)

Factor	All	Subgroup
	(n = 26)	(n = 10)
Sex, male/female	20/6	6/4
Age, mean (SD)	60.2 (9.3)	66.5 (8.6)
BMI, mean (SD)	25.4 (3.2)	24.6 (3.7)
Side, right/left	12/14	4/6

**Figure 1:**
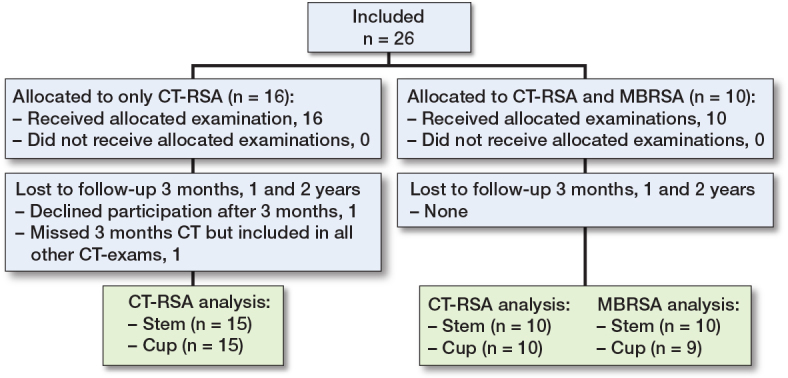
Patient flowchart. MBRSA = model based RSA.

For the AI-based CT-RSA evaluation, 1 patient declined further CT scans after 3 months and thus was excluded. 1 patient missed the 3 months CT but was included in all other examinations. For the MBRSA comparison, 1 patient was excluded from the cup analysis due to difficulties visualizing and identifying beads in several examinations (Figure 1).

### Migration

**Figure 2:**
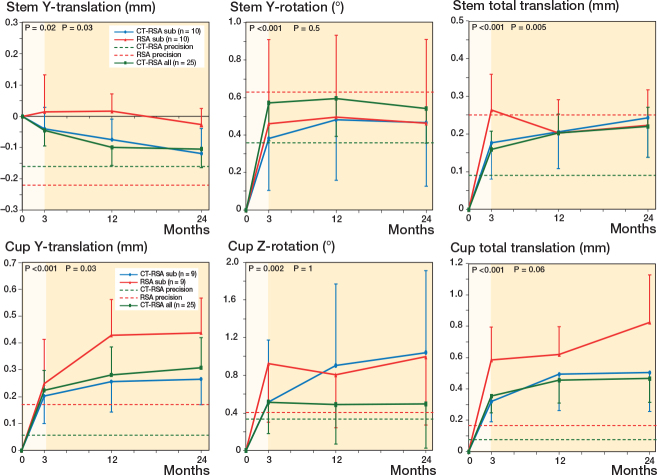
Stem and cup MBRSA and AI-based CT-RSA migration. Mean stem y-translation, y-rotation, and total translation, and cup y-translation, z-rotation, and total translation with 95% confidence interval bars. Blue and red lines represent CT-RSA and MBRSA subgroup analysis with 10 and 9 patients (stem/cup respectively). Green line represent all patients in the study (n = 25) with exclusively AI-based CT-RSA results. Dotted green and red lines are the precision values for AI-based CT-RSA and MBRSA respectively. P-values from linear mixed-effect model for all patients (green line) for slopes 0–3 months (highlighted) and 3–24 months.

Most of the migrations seemed to occur in the first 3 months after surgery, further supported by linear mixed-effect models (Figure 2 and Figure 5, see Supplementary data). After 2 years, the CT-RSA results from all patients showed that the stem had subsided 0.11 mm and the cup had migrated cranially 0.31 mm (y-translation). In addition, the stem retroverted 0.54° (y-rotation) and the cup inclination decreased 0.50° (z-rotation). For patients with both AI-based CT-RSA andMBRSA, the stem subsidence was slightly larger measured with AI-based CT-RSA (0.12 mm) compared with MBRSA (0.03 mm) and the cup had migrated cranially 0.27 mm according to AI-based CT-RSA, compared with 0.44 mm measured with MBRSA 2 years after surgery.

The basis for calculating precision, including mean difference and SD from CT/RSA double exams, is summarized in Table 2. The precision for AI-based CT-RSA was superior for all migrations (Table 2). For AI-based CT-RSA, the translational precision ranged from 0.06–0.16 mm, whereas MBRSA precision ranged from 0.17–0.38 mm. For rotation, the precision ranged between 0.08° and 0.51° for AI-based CT-RSA and 0.24° and 0.66° for MBRSA, respectively.

**Table 2:** Results from double examinations, as basis for calculating precision (described as SD x the critical value of T) of MBRSA (n = 10 cup, n = 12 stem) and AI-based CT-RSA (n = 14), mean and standard deviation (SD) of stem and cup values

Value	Tx	Ty	Tz	Rx	Ry	Rz	Ttot	Rtot
MBRSA								
Stem, mean (SD)	0.02 (0.13)	–0.07 (0.10)	0.06 (0.14)	0.00 (0.22)	–0.03 (0.29)	0.03 (0.11)	0.19 (0.11)	0.34 (0.13)
Precision	0.28	0.22	0.30	0.49	0.63	0.24	0.25	0.29
Cup, mean (SD)	0.03 (0.09)	–0.01 (0.08)	–0.02 (0.17)	–0.10 (0.28)	0.10 (0.30)	0.06 (0.18)	0.19 (0.07)	0.37 (0.27)
Precision	0.19	0.17	0.38	0.62	0.66	0.41	0.17	0.60
AI-based CT-RSA								
Stem, mean (SD)	–0.01 (0.04)	–0.01 (0.07)	0.01 (0.03)	0.01 (0.04)	0.01 (0.17)	0.00 (0.04)	0.08 (0.04)	0.13 (0.11)
Precision	0.08	0.16	0.07	0.08	0.36	0.08	0.09	0.24
Cup, mean (SD)	–0.01 (0.04)	0.00 (0.03)	0.01 (0.04)	–0.03 (0.19)	–0.06 (0.24)	0.02 (0.16)	0.06 (0.04)	0.29 (0.18)
Precision	0.09	0.06	0.09	0.40	0.51	0.34	0.08	0.39

Tx, Ty, Tz, Ttot: Translations (mm).

Rx, Ry, Rz, Rtot: Rotations (°).

**Figure 3:**
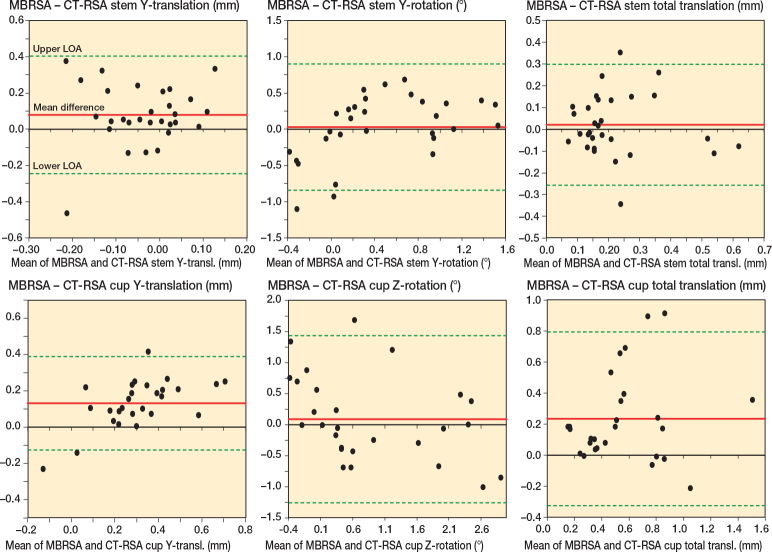
Bland–Altman plots for stem y-translation, y-rotation, and total translation, and cup y-translation, z-rotation, and total translation. Limits of agreement (LoA) are shown as dotted green lines. The mean difference (bias) is shown as a solid red line.

The agreement between MBRSA and AI-based CT-RSA showed a limit of agreement of –0.25 –to 0.40 mm for stem subsidence and –0.13 to 0.39 mm for cup proximal migration (Figure 3) (for all translations and rotations, see Figure 6, see Supplemenntary data). For patients with both AI-based CT-RSA and MBRSA, least square means and difference in least square means with corresponding CIs from repeated measures mixed models are presented in Supplementary Table 3 (see Supplementary data).

### Preoperative 3D planning

Comparing the preoperatively planned (AI suggested but up to the surgeon to adjust) implant size with the one used, there was a 96% (CI 80–100) agreement (25 of 26) between planned and used cup size. For the stem, there was an 88% (CI 70–98) agreement (23 of 26) in stem size, and in 62% (CI 41–80) of cases (16 of 26) there was an exact match between plan and selected, including standard, varus, and high-offset stem options.

### Clinical evaluation

**Figure 4:**
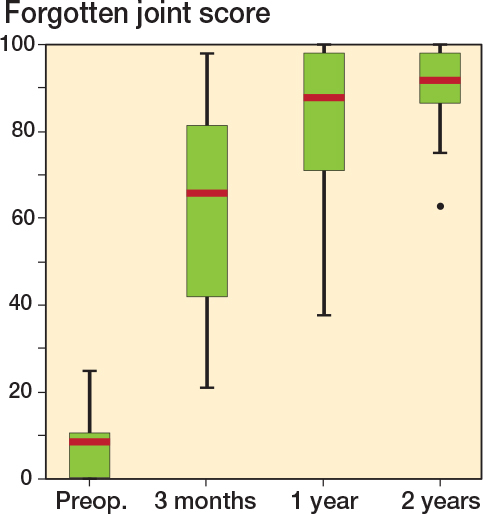
Forgotten joint score (FJS). Scale 0–100, higher scores correlate with a better outcome. Red line is median value.

Patients reported, as expected, a clinical improvement after surgery (Figure 4). The median preoperative FJS score was 8, and 3 months after surgery it was 66. In addition, patient satisfaction continued to improve after surgery at 3–12 months and 12–24 months. At 24 months the score was 92.

## Discussion

To our knowledge, this is the first clinical study on both THA cups and stems comparing CT-RSA with MBRSA with a follow-up program in accordance with RSA guidelines, including double examinations [12]. Our primary goal was to evaluate migration results from the AI-based CT-RSA module of the OTS platform (OTS Follow-up), and in a subgroup directly compare it with MBRSA. Our results show that the cementless stem and cup stabilizes as expected, with an initial, early migration up to the 3rd postoperative month. Thereafter very little additional migration is seen, indicating osseointegration. Moreover, we found good agreement between MBRSA and AI-based CT-RSA in the subgroup analysis, with consistently better precision values for AI-based CT-RSA. Secondarily, the OTS Hip Plan delivered a predictable preoperative implant plan.

Looking at our AI-based CT-RSA migration results for all patients, the Corail stem subsided 0.04 mm after 3 months and 0.11 mm after 2 years with no signs of progressive subsidence (see Figure 2). Other conventional RSA studies on the Corail stem have shown a small settling during the first months, and then stabilization, with 2-year subsidence of about 0.4–0.6 mm [26,27]. A possible explanation for our small migration values compared with previously mentioned studies on both collared and collarless stems could be our exclusive use of stems with collar. 2 years postoperatively, the Pinnacle cup had migrated 0.31 mm proximally measured with AI-based CT-RSA. These results are similar to other conventional RSA studies on uncemented cups [28,29], and within the limit considered acceptable to avoid late loosening (< 1 mm) [30]. It should be noted that we performed the reference examinations within 2 days from surgery, and our migration values thus reflect almost the true complete migration. Some studies carry out the reference examination after 2 weeks or later, even though we know that often about 50% of the 3 months’ migration has already occurred at 2 weeks [31], which may lead to a bias when comparing study results.

In the direct comparison, MBRSA vs AI-based CT-RSA, we find that the results from AI-based CT-RSA look stable with narrower CI than MBRSA (see Figure 2 and Supplementary Figure 5). The AI-based CT-RSA precision, for both cup and stem, was in line with other studies on CT-RSA [13-15] and consistently better than conventional RSA (see Table 2). A similar trend was observed by Brodén et al. with superior CT-RSA rotational precision compared with marker-based RSA [15]. Perhaps this can be attributed to the advantage of 3D registration in CT-RSA, and the possibility of collinear marker distribution affecting the rotational result in conventional RSA.

From the Bland–Altman plot, mean difference between MBRSA and AI-based CT-RSA in stem subsidence was smaller compared with our previously published study [20], suggesting that incorporating the CAD models in AI-based CT-RSA provides more equivalent results to MBRSA. The mean difference in cup proximal translation was similar to our previous findings [20], as well as those from other studies [13]. Furthermore, the results are within the limits of clinical importance of 0.2–1 mm [13] as a predictor of future implant loosening [30]. Overall, we found a higher correspondence between MBRSA and AI-based CT-RSA for translations than for rotations. It should be noted that it is still not clear whether MBRSA or CT-RSA is closest to the true migration. However, as previously mentioned, it is notable that AI-based CT-RSA migration results seems to be more stable, with narrower CI and better precision than conventional RSA. In recent years, CT-RSA has received increasing attention from the international RSA community, and there are now published practical guidelines on how to plan, execute, and publish CT-RSA research [32,33].

A concern with CT-RSA is the high radiation compared with conventional RSA. There has been advancement in low-dose technology in recent years [9], keeping the radiation dose below that of a standard CT pelvis, which is about 6 mSv [34]. The scan protocol was under development and optimization during the study period, and thus there was a spread in the given dose from the beginning to the end of the study. Factors such as large CT slice thickness may negatively affect the accuracy [16,35]. In this study, we did not perform a statistical comparison between the results from those who were given a higher radiation dose and those with a lower dose. Such a comparison would also be difficult to carry out as we do not know the actual true migration. However, dose reduction does not have to negatively affect precision [36], and we could not notice any systematic difference when comparing pelvic segmentation registration accuracy for a few selected cases in the higher/lower radiation spectrum. This indicates that a dose in the lower range (about 1–2 mSv) is probably fully sufficient for the analysis, yet further studies are required on dose optimization.

2D templating in THA typically involves using software for digital radiographs to determine implant size and positioning. Although this method often predicts the implant within 1 size, the exact stem and cup size estimation can be < 50%, with factors such as surgeon experience and patients’ BMI affecting the result [37]. 3D templating enables detailed reconstruction of the patient’s anatomy, demonstrating greater accuracy than 2D [38-40]. For inexperienced surgeons, AI has been shown to be a valuable adjunctive tool for the preoperative planning [41]. In our study, there was a > 85% agreement between planned and resulting implant size. These results were superior, or in line with previous studies on predicting implant size in 3D [40,42,43]; however, we would have needed a larger cohort to draw any final conclusions.

### Limitations

For the MBRSA vs AI-based CT-RSA comparison, we only have 10 of the 26 patients included, in order to keep the radiation exposure limited. Patients in the MBRSA subgroup were slightly older (66.5 years) than the entire cohort (60.2 years), but otherwise demographically similar (see Table 1).

Another limitation was the inconsistency in low-dose CT protocols. The CT examinations were done on different scanners, and the protocols were under development with different settings and various radiation levels as a result.

Using CAD models for both AI-based CT-RSA and conventional RSA makes the comparison more equal; however, sometimes implant CAD models are not easily provided by the manufacturer and thus are considered as a limitation. This AI-based CT-RSA software can, however, be used without CAD models [20].

Furthermore, we believe AI-based CT-RSA stem rotation values can be improved. The software is currently only using a stem CAD model for the analysis. By including the head of the stem, we believe that, above all, stem ante-/retroversion (y-rotation) will be improved.

### Conclusion

We showed good agreement between AI-based CT-RSA and MBRSA. CT-based 3D-planning accurately predicted implant size and type.

*In perspective*, CT-RSA seems to be as a feasible alternative to MBRSA including stable results, and potentially better CT-RSA precision. In addition, implementing AI makes it user-independent, faster, and more accessible.

## Supplementary Material



## Supplementary data

Supplementary Table 3 and Figures 5–6 are available as supplementary data on the article page, doi: 10.2340/17453674.2025.44948

AC: Compiled, analyzed, and organized the data. Participated in writing the manuscript. HN: Performing the AI-based CT-RSA software migration analyses and organizing the CT-migration data. Actively participated in the research, revision of manuscript, and approved the article for submission. GF: Study design. Performing operations. Actively participated in the research, revision of manuscript, and approved the article for submission. KY: Development and updating the CT scanning protocol, analyzing and compiling radiological data, revision, and approving the manuscript for submission.

The authors would like to thank Håkan Leijon at the RSA Laboratory, Skåne University Hospital, for help in computerizing the RSA images, and research secretary Åsa Björkqvist, Department of Orthopedics, Skåne University Hospital, for keeping track of all the patients. Further thanks are offered to Tommy Schyman, Biostatistician at BC Platforms, Lund, Sweden, for statistical advice.

Handling co-editors: Ivan Hvid and Robin Christensen

*Acta* thanks Petra Heesterbeek and Stephan Maximilian Röhrl for help with peer review of this manuscript.
