# Supplementary material for: Evaluation of migration analysis with AI-based CT-RSA and preoperative 3D-planning in total hip arthroplasty
Source: Acta Orthop. 2025 Dec 11;96:885–92. doi: 10.2340/17453674.2025.44948 (PMC12697192; doi:10.2340/17453674.2025.44948)
Supplement: Supplementary file 1 [file ActaO-96-44948-s1.pdf]

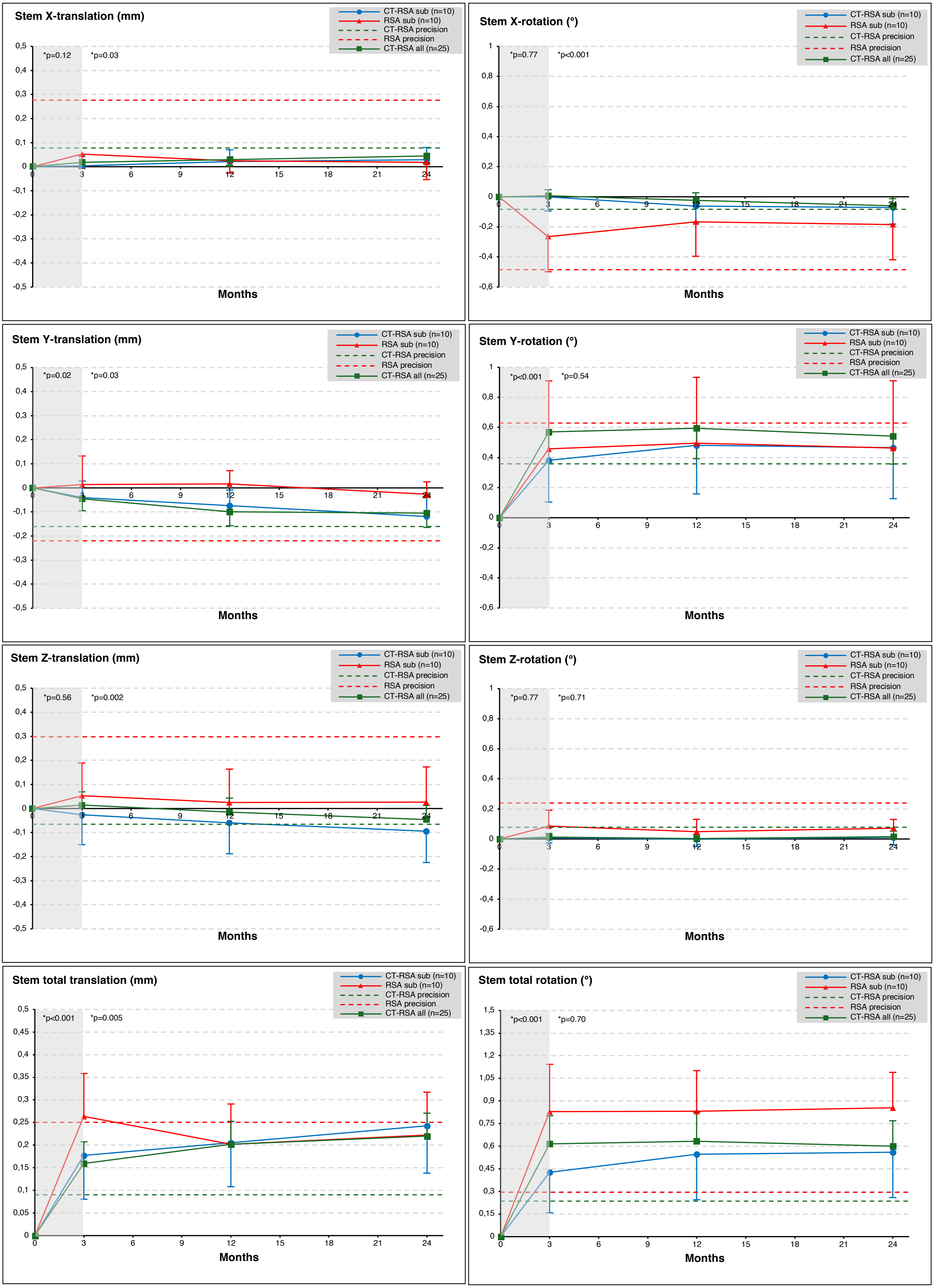

Stem X-translation (mm)
*p=0.12
*p=0.03
Stem X-rotation (°)
*p=0.77
*p<0.001
Stem Y-translation (mm)
*p=0.02
*p=0.03
Stem Y-rotation (°)
*p<0.001
*p=0.54
Stem Z-translation (mm)
*p=0.56
*p=0.002
Stem Z-rotation (°)
*p=0.77
*p=0.71
Stem total translation (mm)
*p<0.001
*p=0.005
Stem total rotation (°)
*p<0.001
*p=0.70
CT-RSA sub (n=10)
RSA sub (n=10)
CT-RSA precision
RSA precision
CT-RSA all (n=25)
Months

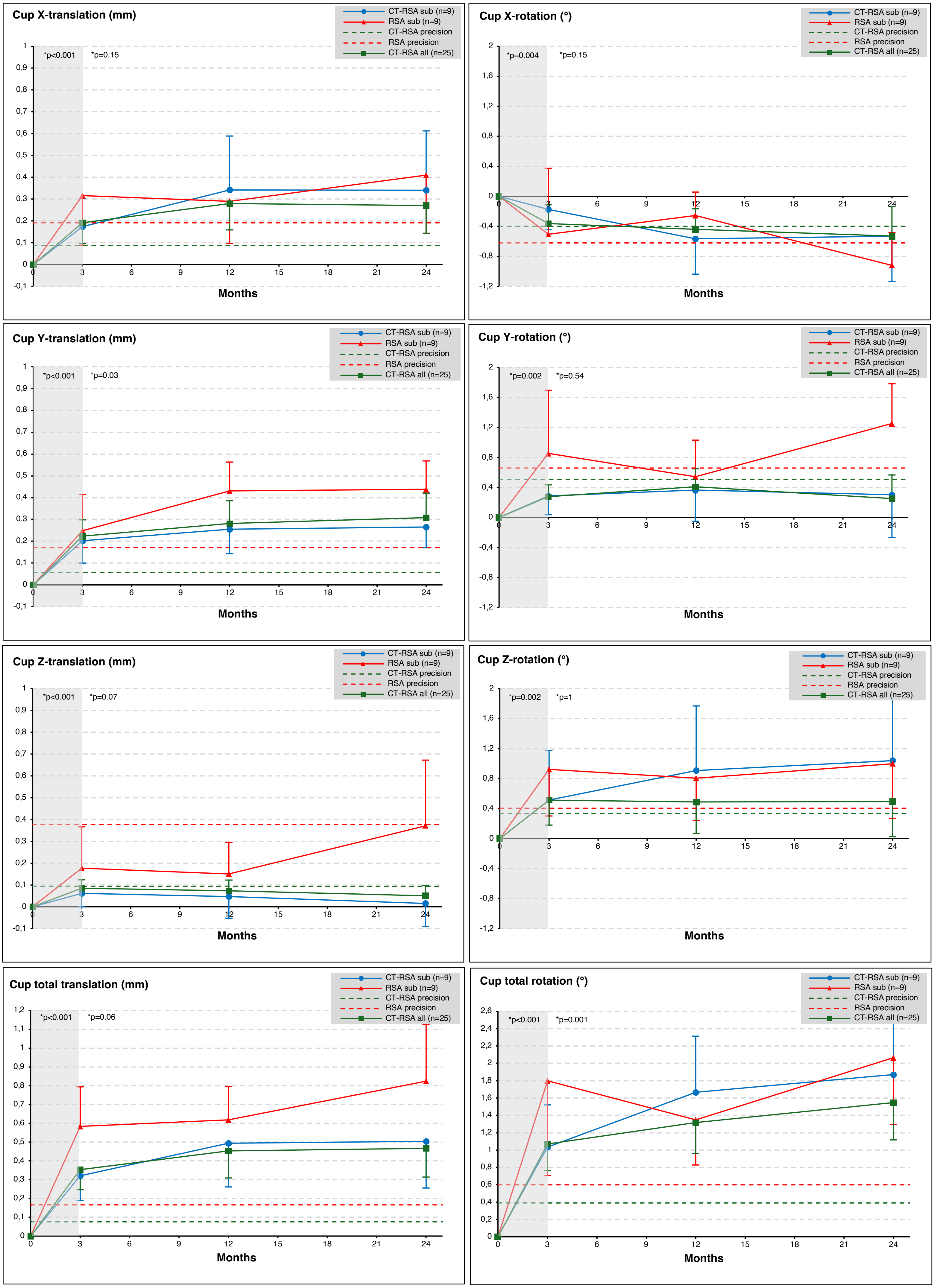
Cup X-translation (mm)
CT-RSA sub (n=9)
RSA sub (n=9)
CT-RSA precision
RSA precision
CT-RSA all (n=25)
*p<0.001
*p=0.15
Months
Cup X-rotation (°)
*p=0.004
*p=0.15
Months
Cup Y-translation (mm)
*p<0.001
*p=0.03
Months
Cup Y-rotation (°)
*p=0.002
*p=0.54
Months
Cup Z-translation (mm)
*p<0.001
*p=0.07
Months
Cup Z-rotation (°)
*p=0.002
*p=1
Months
Cup total translation (mm)
*p<0.001
*p=0.06
Months
Cup total rotation (°)
*p<0.001
*p=0.001
Months

Supplementary data Figure 5.
Stem and cup MBRSA and AI-based CT-RSA migration. Mean x-, y-, z-translations/rotations, total translation and rotation with 95% CI bars. Blue and red lines representing CT-RSA and MBRSA subgroup analysis with 10 and 9 patients (stem resp cup). Green line representing all patents in the study (n=25) with exclusively AI-based CT-RSA results. Dotted green and red lines are the precision values for AI-based CT-RSA and MBRSA respectively. Months 0-3 highlighted in light grey.

*P-values from linear mixed-effect model for all patients (green line). Slopes 0-3 months and 3-24 months.

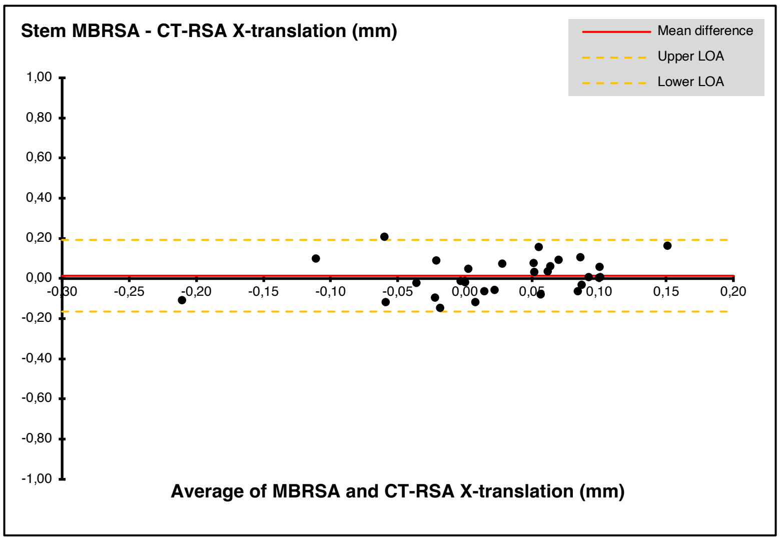
Stem MBRSA - CT-RSA X-translation (mm)
Mean difference
Upper LOA
Lower LOA
1,00 0,80 0,60 0,40 0,20 0,00 -0,20 -0,40 -0,60 -0,80 -1,00
-0,30 -0,25 -0,20 -0,15 -0,10 -0,05 0,00 0,05 0,10 0,15 0,20
Average of MBRSA and CT-RSA X-translation (mm)

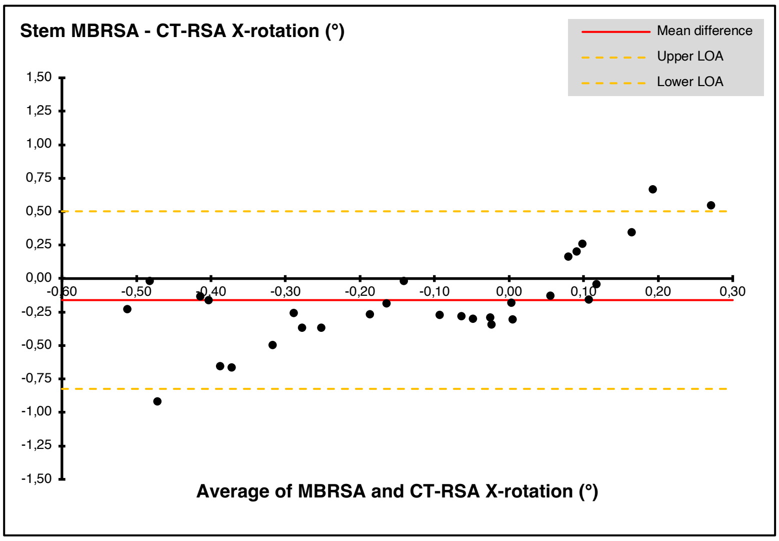
Stem MBRSA - CT-RSA X-rotation (°)
Mean difference
Upper LOA
Lower LOA
1,50 1,25 1,00 0,75 0,50 0,25 0,00 -0,25 -0,50 -0,75 -1,00 -1,25 -1,50
-0,60 -0,50 -0,40 -0,30 -0,20 -0,10 0,00 0,10 0,20 0,30
Average of MBRSA and CT-RSA X-rotation (°)

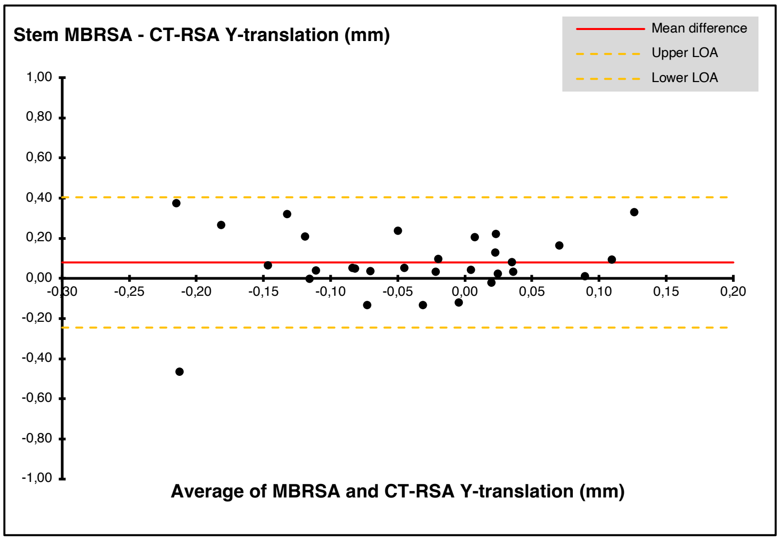
Stem MBRSA - CT-RSA Y-translation (mm)
Mean difference
Upper LOA
Lower LOA
1,00 0,80 0,60 0,40 0,20 0,00 -0,20 -0,40 -0,60 -0,80 -1,00
-0,30 -0,25 -0,20 -0,15 -0,10 -0,05 0,00 0,05 0,10 0,15 0,20
Average of MBRSA and CT-RSA Y-translation (mm)

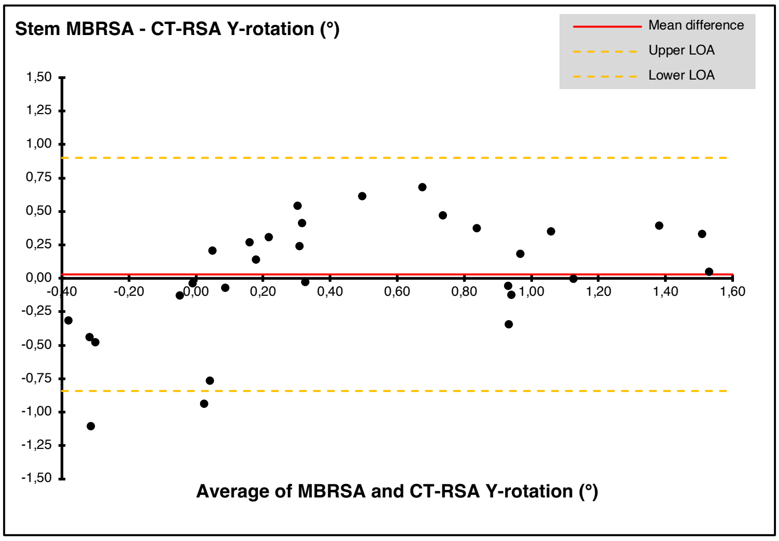
Stem MBRSA - CT-RSA Y-rotation (°)
Mean difference
Upper LOA
Lower LOA
1,50 1,25 1,00 0,75 0,50 0,25 0,00 -0,25 -0,50 -0,75 -1,00 -1,25 -1,50
-0,40 -0,20 0,00 0,20 0,40 0,60 0,80 1,00 1,20 1,40 1,60
Average of MBRSA and CT-RSA Y-rotation (°)

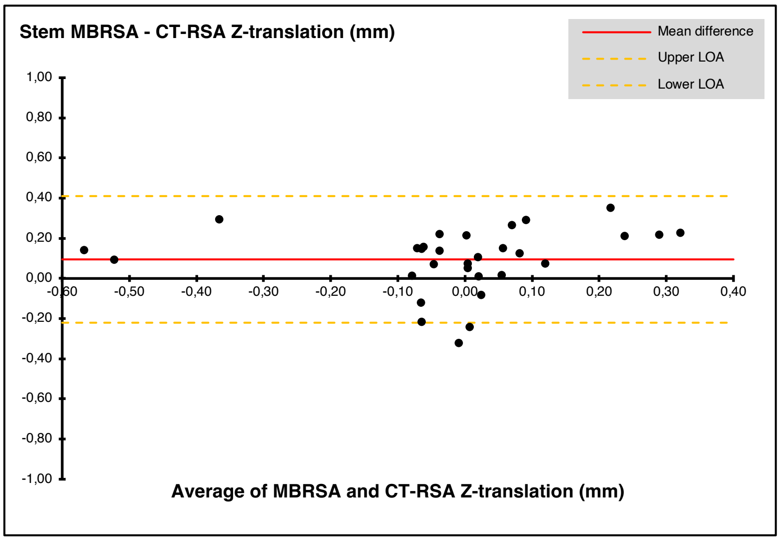
Stem MBRSA - CT-RSA Z-translation (mm)
Mean difference
Upper LOA
Lower LOA
1,00 0,80 0,60 0,40 0,20 0,00 -0,20 -0,40 -0,60 -0,80 -1,00
-0,60 -0,50 -0,40 -0,30 -0,20 -0,10 0,00 0,10 0,20 0,30 0,40
Average of MBRSA and CT-RSA Z-translation (mm)

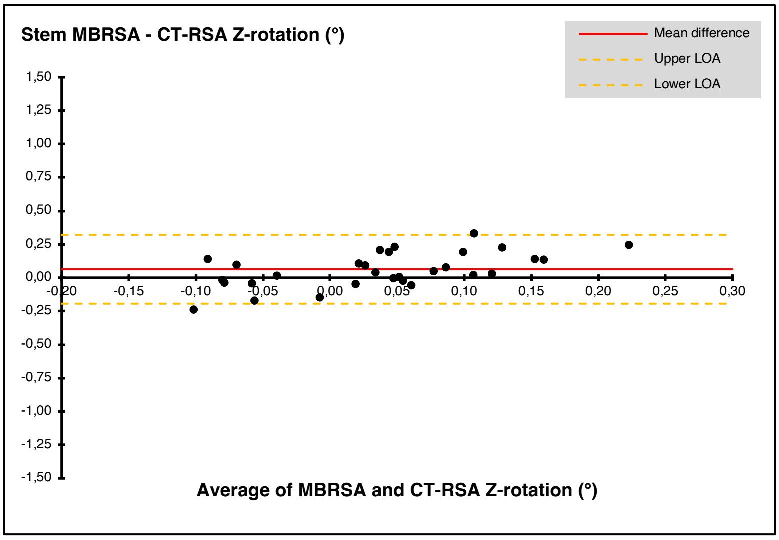
Stem MBRSA - CT-RSA Z-rotation (°)
Mean difference
Upper LOA
Lower LOA
1,50 1,25 1,00 0,75 0,50 0,25 0,00 -0,25 -0,50 -0,75 -1,00 -1,25 -1,50
-0,20 -0,15 -0,10 -0,05 0,00 0,05 0,10 0,15 0,20 0,25 0,30
Average of MBRSA and CT-RSA Z-rotation (°)

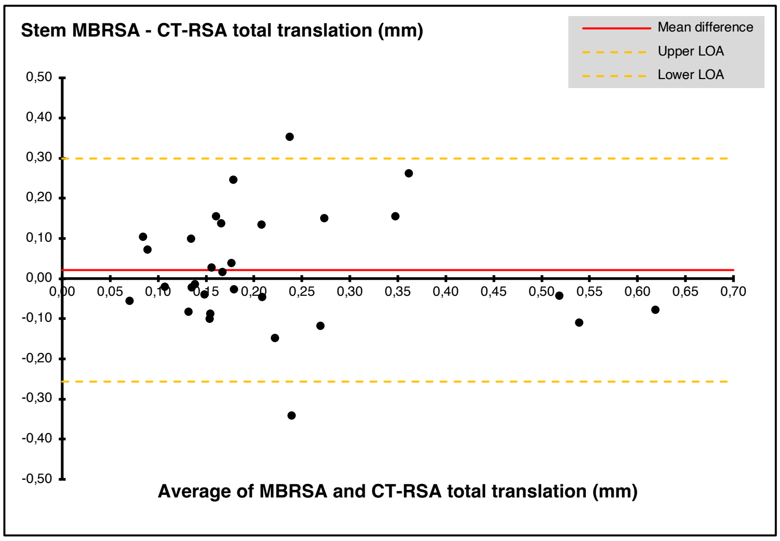
Stem MBRSA - CT-RSA total translation (mm)
Mean difference
Upper LOA
Lower LOA
0,50 0,40 0,30 0,20 0,10 0,00 -0,10 -0,20 -0,30 -0,40 -0,50
0,00 0,05 0,10 0,15 0,20 0,25 0,30 0,35 0,40 0,45 0,50 0,55 0,60 0,65 0,70
Average of MBRSA and CT-RSA total translation (mm)

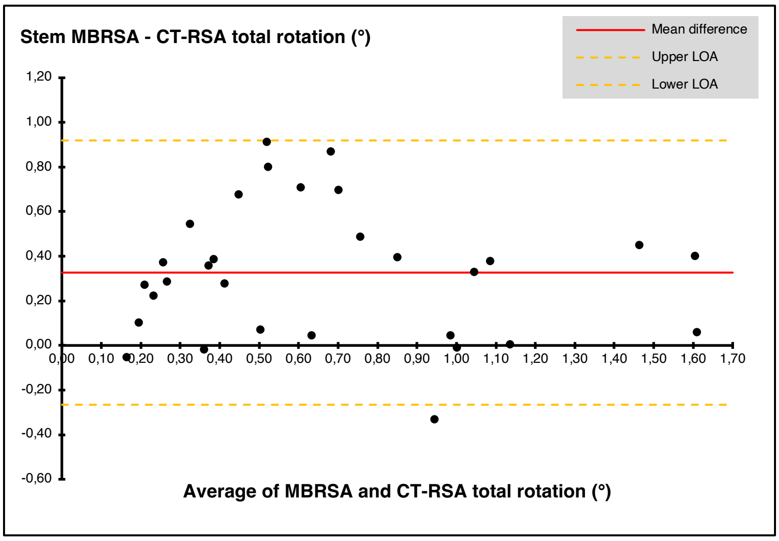
Stem MBRSA - CT-RSA total rotation (°)
Mean difference
Upper LOA
Lower LOA
1,20 1,00 0,80 0,60 0,40 0,20 0,00 -0,20 -0,40 -0,60
0,00 0,10 0,20 0,30 0,40 0,50 0,60 0,70 0,80 0,90 1,00 1,10 1,20 1,30 1,40 1,50 1,60 1,70
Average of MBRSA and CT-RSA total rotation (°)

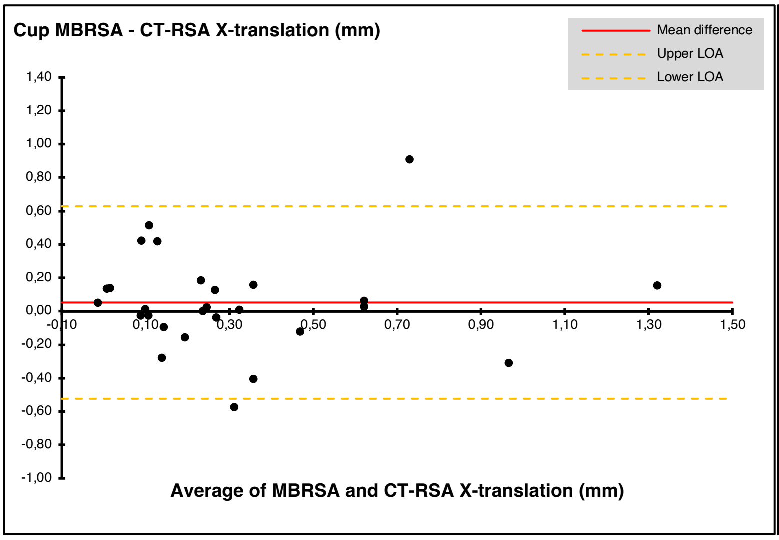
Cup MBRSA - CT-RSA X-translation (mm)
Mean difference
Upper LOA
Lower LOA
Average of MBRSA and CT-RSA X-translation (mm)

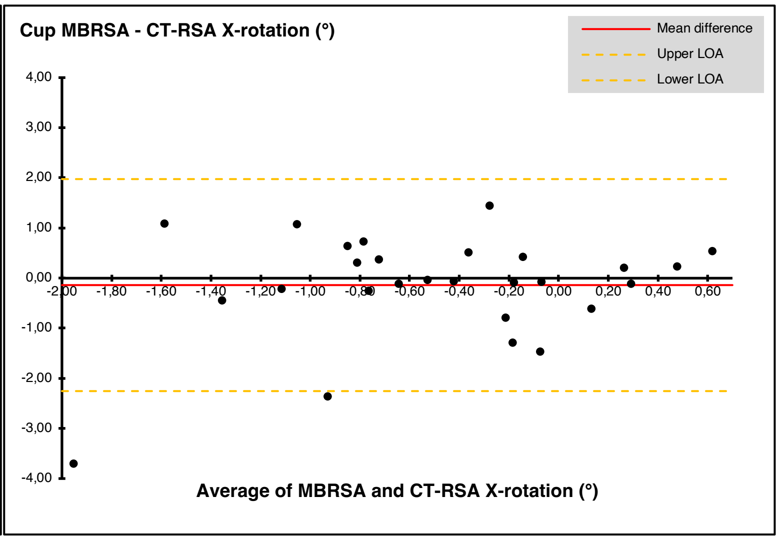
Cup MBRSA - CT-RSA X-rotation (°)
Mean difference
Upper LOA
Lower LOA
Average of MBRSA and CT-RSA X-rotation (°)

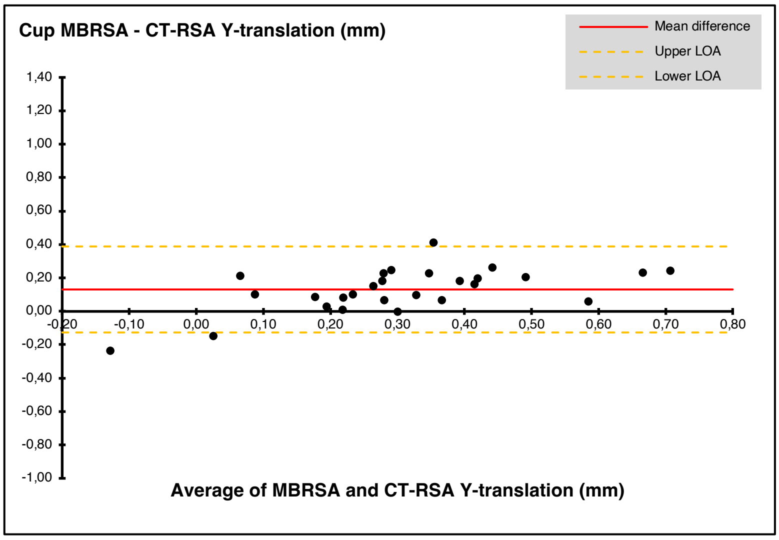
Cup MBRSA - CT-RSA Y-translation (mm)
Mean difference
Upper LOA
Lower LOA
Average of MBRSA and CT-RSA Y-translation (mm)

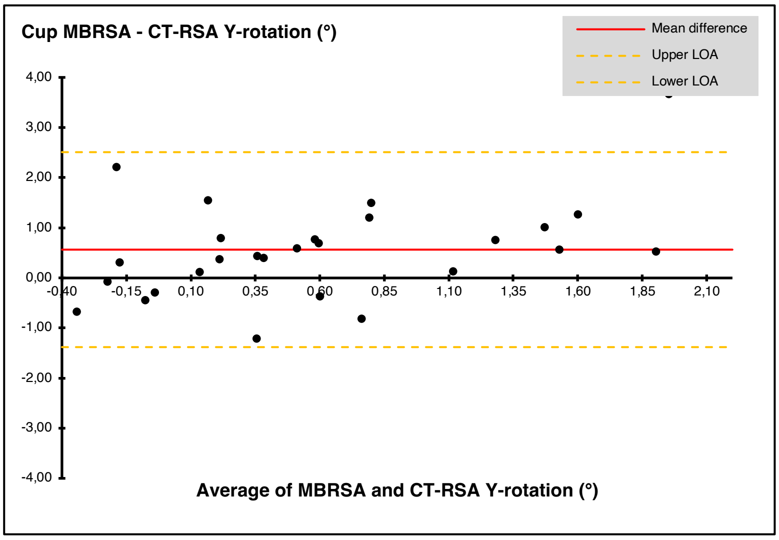
Cup MBRSA - CT-RSA Y-rotation (°)
Mean difference
Upper LOA
Lower LOA
Average of MBRSA and CT-RSA Y-rotation (°)

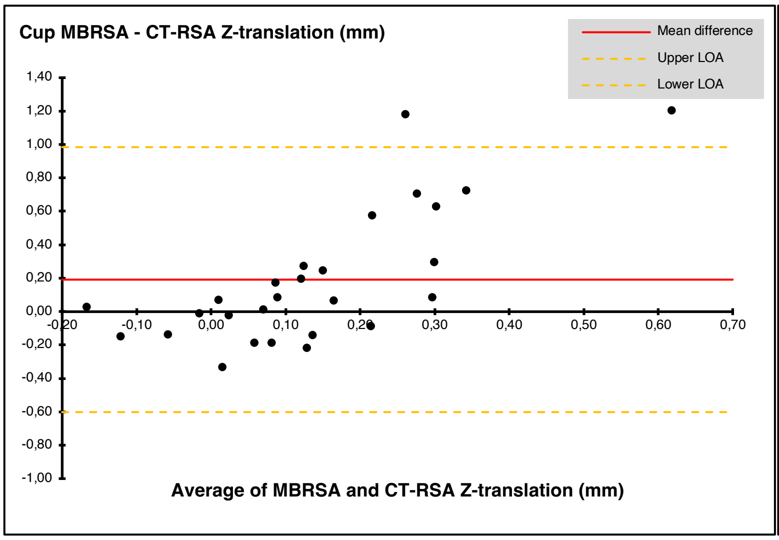
Cup MBRSA - CT-RSA Z-translation (mm)
Mean difference
Upper LOA
Lower LOA
Average of MBRSA and CT-RSA Z-translation (mm)

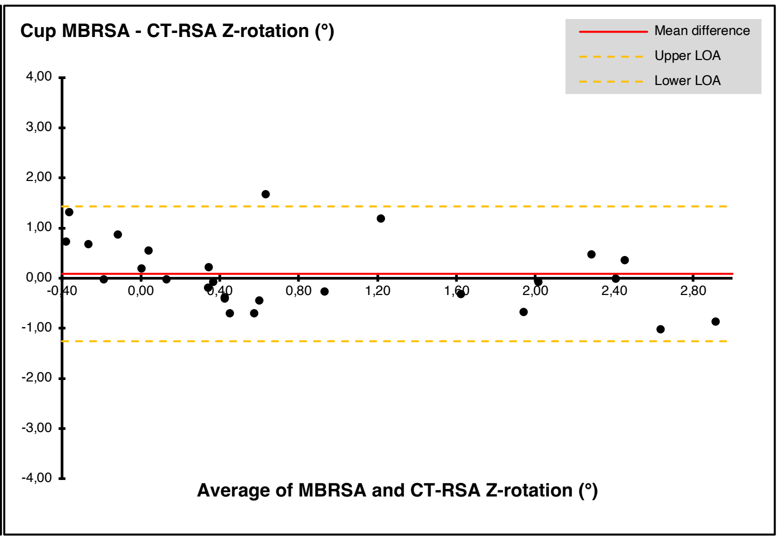
Cup MBRSA - CT-RSA Z-rotation (°)
Mean difference
Upper LOA
Lower LOA
Average of MBRSA and CT-RSA Z-rotation (°)

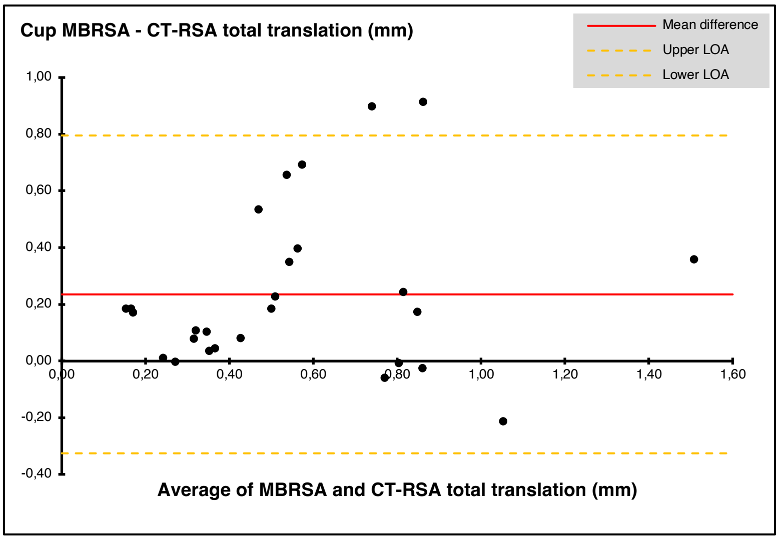
Cup MBRSA - CT-RSA total translation (mm)
Mean difference
Upper LOA
Lower LOA
Average of MBRSA and CT-RSA total translation (mm)

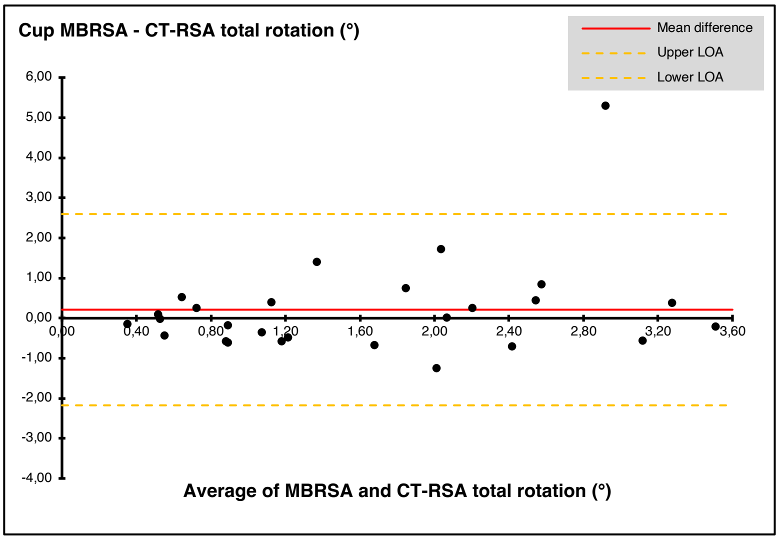
Cup MBRSA - CT-RSA total rotation (°)
Mean difference
Upper LOA
Lower LOA
Average of MBRSA and CT-RSA total rotation (°)

Supplementary data Figure 6.

Bland–Altman plots for stem and cup x-, y-, z-translations/rotations, total translation and rotation. Limits of agreement (LoA) are shown as dotted yellow lines. The mean difference (bias) is shown as a solid red line.

Supplementary data Table 3A. Repeated measures mixed models including the 10 subjects having both CT-RSA and RSA, cup results. Least square means and difference in least square means with corresponding 95% confidence intervals for 3, 12 and 24 months after surgery.

				Least square mean (95% CI)		
**Part**	**Axis**	**Migration**	**Month**	**CT-RSA**	**RSA**	**LS mean difference (95% CI)**
**Cup**	**Total**	**Rotation**	3	1.342 (0.686 to 1.998)	1.709 (1.032 to 2.386)	-0.367 (-1.050 to 0.316)
			12	1.711 (1.137 to 2.285)	1.846 (1.261 to 2.432)	-0.135 (-0.587 to 0.318)
			24	2.204 (1.530 to 2.877)	2.029 (1.333 to 2.725)	0.175 (-0.548 to 0.897)
**Cup**	**Total**	**Translation**	3	0.436 (0.230 to 0.643)	0.600 (0.388 to 0.811)	-0.163 (-0.347 to 0.020)
			12	0.501 (0.311 to 0.691)	0.705 (0.513 to 0.898)	-0.204 (-0.326 to -0.083)
			24	0.587 (0.377 to 0.798)	0.847 (0.631 to 1.062)	-0.259 (-0.454 to -0.065)
**Cup**	**X**	**Rotation**	3	-0.401 (-0.883 to 0.081)	-0.432 (-0.933 to 0.069)	0.031 (-0.513 to 0.575)
			12	-0.583 (-0.991 to -0.175)	-0.628 (-1.047 to -0.209)	0.045 (-0.315 to 0.405)
			24	-0.825 (-1.323 to -0.327)	-0.889 (-1.406 to -0.371)	0.064 (-0.511 to 0.639)
**Cup**	**X**	**Translation**	3	0.274 (0.075 to 0.473)	0.323 (0.120 to 0.527)	-0.049 (-0.227 to 0.129)
			12	0.332 (0.150 to 0.514)	0.366 (0.182 to 0.551)	-0.034 (-0.152 to 0.084)
			24	0.409 (0.207 to 0.612)	0.424 (0.216 to 0.631)	-0.014 (-0.203 to 0.174)
**Cup**	**Y**	**Rotation**	3	0.319 (-0.114 to 0.752)	0.641 (0.191 to 1.092)	-0.322 (-0.829 to 0.184)
			12	0.258 (-0.098 to 0.614)	0.832 (0.464 to 1.199)	-0.574 (-0.909 to -0.238)
			24	0.177 (-0.272 to 0.625)	1.085 (0.618 to 1.553)	-0.909 (-1.445 to -0.373)
**Cup**	**Y**	**Translation**	3	0.266 (0.135 to 0.398)	0.320 (0.186 to 0.454)	-0.054 (-0.156 to 0.049)
			12	0.290 (0.166 to 0.414)	0.397 (0.272 to 0.523)	-0.107 (-0.175 to -0.040)
			24	0.322 (0.188 to 0.455)	0.501 (0.365 to 0.636)	-0.179 (-0.287 to -0.071)
**Cup**	**Z**	**Rotation**	3	0.797 (0.116 to 1.478)	1.009 (0.317 to 1.702)	-0.212 (-0.746 to 0.321)
			12	1.014 (0.374 to 1.655)	1.047 (0.401 to 1.693)	-0.033 (-0.387 to 0.321)
			24	1.304 (0.614 to 1.994)	1.098 (0.395 to 1.800)	0.207 (-0.358 to 0.771)
**Cup**	**Z**	**Translation**	3	0.073 (-0.050 to 0.196)	0.137 (0.007 to 0.267)	-0.064 (-0.235 to 0.107)
			12	0.051 (-0.036 to 0.139)	0.225 (0.133 to 0.317)	-0.173 (-0.286 to -0.060)
			24	0.022 (-0.107 to 0.152)	0.341 (0.204 to 0.478)	-0.319 (-0.500 to -0.138)

Supplementary data Table 3B. Repeated measures mixed models including the 10 subjects having both CT-RSA and RSA, stem results. Least square means and difference in least square means with corresponding 95% confidence intervals for 3, 12 and 24 months after surgery.

				Least square mean (95% CI)		
**Part**	**Axis**	**Migration**	**Month**	**CT-RSA**	**RSA**	**LS mean difference (95% CI)**
**Stem**	**Total**	**Rotation**	3	0.451 (0.201 to 0.701)	0.825 (0.575 to 1.075)	-0.374 (-0.574 to -0.174)
			12	0.505 (0.273 to 0.738)	0.837 (0.604 to 1.069)	-0.331 (-0.463 to -0.200)
			24	0.578 (0.324 to 0.832)	0.853 (0.599 to 1.107)	-0.274 (-0.486 to -0.063)
**Stem**	**Total**	**Translation**	3	0.177 (0.093 to 0.261)	0.247 (0.163 to 0.331)	-0.070 (-0.144 to 0.004)
			12	0.205 (0.129 to 0.281)	0.231 (0.155 to 0.307)	-0.026 (-0.074 to 0.022)
			24	0.243 (0.157 to 0.328)	0.210 (0.125 to 0.296)	0.032 (-0.045 to 0.110)
**Stem**	**X**	**Rotation**	3	-0.013 (-0.163 to 0.138)	-0.241 (-0.392 to -0.091)	0.229 (0.073 to 0.384)
			12	-0.041 (-0.171 to 0.089)	-0.209 (-0.339 to -0.079)	0.168 (0.066 to 0.270)
			24	-0.080 (-0.235 to 0.075)	-0.166 (-0.321 to -0.011)	0.086 (-0.078 to 0.251)
**Stem**	**X**	**Translation**	3	0.006 (-0.040 to 0.051)	0.047 (0.002 to 0.093)	-0.042 (-0.091 to 0.007)
			12	0.016 (-0.022 to 0.055)	0.033 (-0.005 to 0.072)	-0.017 (-0.049 to 0.015)
			24	0.031 (-0.016 to 0.078)	0.014 (-0.032 to 0.061)	0.016 (-0.035 to 0.068)
**Stem**	**Y**	**Rotation**	3	0.407 (0.068 to 0.746)	0.473 (0.133 to 0.812)	-0.066 (-0.341 to 0.209)
			12	0.440 (0.125 to 0.754)	0.473 (0.158 to 0.787)	-0.033 (-0.214 to 0.148)
			24	0.484 (0.139 to 0.828)	0.473 (0.128 to 0.818)	0.011 (-0.281 to 0.302)
**Stem**	**Y**	**Translation**	3	-0.041 (-0.102 to 0.021)	0.022 (-0.040 to 0.083)	-0.062 (-0.137 to 0.013)
			12	-0.074 (-0.122 to -0.026)	0.003 (-0.044 to 0.051)	-0.078 (-0.127 to -0.028)
			24	-0.119 (-0.183 to -0.055)	-0.021 (-0.085 to 0.043)	-0.098 (-0.178 to -0.019)
**Stem**	**Z**	**Rotation**	3	0.003 (-0.053 to 0.059)	0.074 (0.019 to 0.130)	-0.071 (-0.133 to -0.010)
			12	0.005 (-0.041 to 0.052)	0.070 (0.023 to 0.116)	-0.064 (-0.105 to -0.024)
			24	0.008 (-0.049 to 0.066)	0.063 (0.006 to 0.120)	-0.055 (-0.120 to 0.010)
**Stem**	**Z**	**Translation**	3	-0.028 (-0.145 to 0.089)	0.047 (-0.070 to 0.165)	-0.075 (-0.173 to 0.022)
			12	-0.057 (-0.165 to 0.051)	0.036 (-0.072 to 0.145)	-0.093 (-0.157 to -0.029)
			24	-0.095 (-0.215 to 0.024)	0.022 (-0.098 to 0.141)	-0.117 (-0.220 to -0.014)

LS: Least square

Difference is calculated as CT-RSA - RSA
